# Pre-hospital treatment of acute poisonings in Oslo

**DOI:** 10.1186/1471-227X-8-15

**Published:** 2008-11-24

**Authors:** Fridtjof Heyerdahl, Knut E Hovda, Mari A Bjornaas, Anne K Nore, Jose CP Figueiredo, Oivind Ekeberg, Dag Jacobsen

**Affiliations:** 1 Department of Acute Medicine, Ullevaal University Hospital, Oslo, Norway; 2 Department of Behavioural Sciences in Medicine, Institute of Basic Medical Sciences, Faculty of Medicine, University of Oslo, Norway; 3 Oslo Emergency Ward, Oslo, Norway; 4 Pre-hospital Division, Ullevaal University Hospital, Oslo, Norway

## Abstract

**Background:**

Poisoned patients are often treated in and discharged from pre-hospital health care settings. Studies of poisonings should therefore not only include hospitalized patients. Aims: To describe the acutely poisoned patients treated by ambulance personnel and in an outpatient clinic; compare patients transferred to a higher treatment level with those discharged without transfer; and study the one-week mortality after pre-hospital discharge.

**Methods:**

A one-year multi-centre study with prospective inclusion of all acutely poisoned patients ≥ 16 years of age treated in ambulances, an outpatient clinic, and hospitals in Oslo.

**Results:**

A total of 3757 health service contacts from 2997 poisoning episodes were recorded: 1860 were treated in ambulances, of which 15 died and 750 (40%) were discharged without transfer; 956 were treated in outpatient clinic, of which 801 (84%) were discharged without transfer; and 941 episodes were treated in hospitals. Patients discharged alive after ambulance treatment were mainly poisoned by opiates (70%), were frequently comatose (35%), had respiratory depression (37%), and many received naloxone (49%). The majority of the patients discharged from the outpatient clinic were poisoned by ethanol (55%), fewer were comatose (10%), and they rarely had respiratory depression (4%). Among the hospitalized, pharmaceutical poisonings were most common (58%), 23% were comatose, and 7% had respiratory depression. Male patients comprised 69% of the pre-hospital discharges, but only 46% of the hospitalized patients. Except for one patient, who died of a new heroin overdose two days following discharge from an ambulance, there were no deaths during the first week after the poisonings in the 90% of the pre-hospital discharged patients with known identity.

**Conclusion:**

More than half of the poisoned patients treated in pre-hospital treatment settings were discharged without transfer to higher levels. These poisonings were more often caused by drug and alcohol abuse than in those who were hospitalized, and more than two-thirds were males. Almost half of those discharged from ambulances received an antidote. The pre-hospital treatment of these poisonings appears safe regarding short-term mortality.

## Background

Poisoned patients are not treated in emergency departments and hospitals only [1-3]. In Scandinavia, poisonings related to drug abuse, especially heroin overdoses, are frequently discharged from the ambulance service without admission to hospital. Pre-hospital discharge may be due to the patients' refusal to be admitted, but can also be due to a medical judgement made by the paramedics or physicians on site. The number of patients discharged from ambulances and the outpatient clinic is difficult to determine because these patients are not recorded in hospital records, and the ambulance record databases may contain inaccurate diagnoses.

Most studies of acute poisonings focus on hospital admissions or emergency department visits, either using hospital records or by prospective inclusion of patients; but with no inclusion of poisonings treated outside hospitals. Studies based on poison information centre registers seldom give precise information regarding the poisonings [4,5]. To study the epidemiology of all acute poisonings in a geographically defined area, prospective inclusion of patients in all health care facilities at the different health care levels treating such patients is required. To our knowledge, no such study has previously been performed.

We performed a study on all acute poisonings independent of treatment levels before discharge, with the following aims: 1) to describe the acutely poisoned patients treated by ambulance personnel and an outpatient clinic; 2) to compare patients transferred to a higher treatment level with patients discharged without transfer, and 3) to study one-week mortality after pre-hospital discharge.

## Methods

### Study design

This was a one-year multi-centre study with prospective inclusion of patients. The inclusion criteria were patients ≥ 16 years of age with a main diagnosis of acute poisoning, either intentional or unintentional, who were treated by the Oslo Ambulance Service, Oslo Emergency Ward (outpatient clinic, not hospital-based) or in one of the four emergency hospitals in Oslo. Exclusion criteria were chronic poisoning and patients with another primary diagnosis such as trauma, even if there was an additional acute poisoning. The hospitalized population has been described previously [6,7].

### The medical emergency system in Oslo

The medical emergency system in Oslo is relatively simple and clear, with only one ambulance service (part of the public hospital system); one single large outpatient clinic (Oslo Emergency Ward, not hospital-based) located in central downtown; and all patients requiring admission are transferred to one of the four public emergency hospitals. These institutions cover the medical treatment of all acute poisonings in Oslo. Rare exceptions may be cases treated by general practitioners without transfer to a higher health care level or cases admitted directly to departments not specialized in internal medicine. Only one ambulance carry an emergency physician, which is used for severe medical emergencies of all kind. All other units are led by ambulance officers, who work relatively independent regarding the clinical evaluation and treatment on site. The ambulance officers decide transfer to a higher treatment level or discharge on site based on written guidelines and their own clinical judgement. All patients with unstable conditions or in need for specific treatment are transported to hospital, while stable patients in need for longer observation than possible on scene (about 10–20 minutes) are transported to the outpatient clinic.

### Study sample

**Figure 1:**
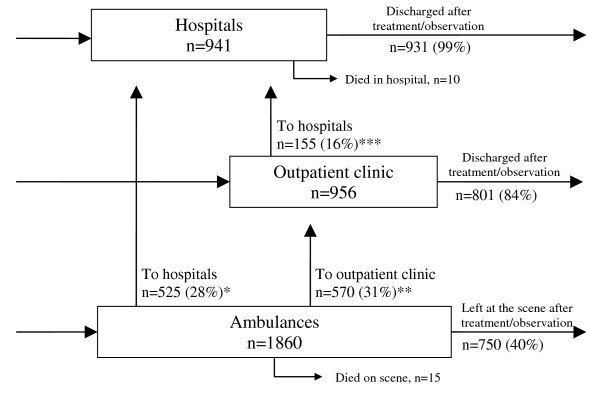
**Acute poisonings treated at three health care levels in Oslo during one year**. In total, there were 3757 health service contacts (3774 including double contacts the same day) from 2997 poisoning episodes by 2298 individuals. *From the 525 transfers between the ambulances and the hospitals, 15 went to hospitals outside Oslo, 15 had missing identity, 98 were not included in the study at the hospitals for various reasons and 397 were included in the study at the receiving hospital. **From the 570 transfers between ambulances and the outpatient clinic, 54 had missing identity, 249 were not included in the study at the outpatient clinic for various reasons and 267 were included in the study at the outpatient clinic. ***From the 155 transfers between the outpatient clinic and the hospitals, 19 went to hospitals outside Oslo, two had missing identity, 38 were not included in the study at the hospitals for various reasons and 96 were included in the study at the receiving hospital.

There were a total of 3774 health service contacts during one year (Figure 1). Because more than one episode during the same day was considered as one episode (n = 17), 3757 contacts was the number used. Because of transfers, more than one health service contact for each poisoning episode was possible, and these contacts resulted from 2997 poisoning episodes in 2298 individuals.

In all, there were 236 (10%) persons with missing or uncertain identity in the study: four of those died in ambulances; two were transferred to hospitals outside Oslo; 10 were admitted to hospitals in Oslo; 71 were transferred from the outpatient clinic or ambulance; and 149 were discharged from ambulances or the outpatient clinic.

In all, 505 (17%) episodes were excluded from the comparisons between levels of care: 71 episodes with unknown identity transferred from a pre-hospital setting, and hence impossible to trace; 385 (13%) episodes with known identities registered for transfer to a higher health care level, but not registered at the receiving institution; 34 episodes transferred to hospitals outside Oslo; and 15 who died in the ambulance.

To investigate what happened to the transferred cases who were lost to follow-up (n = 385), we retrospectively checked a random sample of these medical records in the hospitals (n = 40) and the outpatient clinic (n = 30): 42% were diagnosed with another main diagnosis (in addition to the poisoning) in the receiving institution, and hence not included due to the inclusion criteria (typically opiate overdoses given naloxone by paramedics and brought to the outpatient clinic for the stitching of a wound, or other cases when the main clinical condition changes from poisoning to other problems); 23% were never seen at the receiving institution, probably because they walked away; 20% were acute poisonings that should have been included in the study; 4% were not poisonings at all; and 10% were due to miscellaneous other causes.

Some cases in the hospitals were not registered in the pre-hospital levels. This may have occurred when the ambulances served only as transport, transferring from general practitioners or other physicians to hospitals without providing any independent treatment or diagnostic tasks. Some of the hospitalized patients were also brought by private transport, the police or other means. Further, unclear conditions were sometimes not diagnosed as poisonings before admitted to hospital, where the diagnosis was obtained.

### Data collection

Data were collected from 1 April 2003 until 31 March 2004. In order to ensure complete collection of data, all participating centres had a study coordinator ensuring that all eligible patients were included, and the centres were followed up on a weekly basis by the researchers. Standardized registration forms were completed by physicians in the hospitals and the outpatient clinic and by paramedics in the ambulance service. The forms were optically scanned and processed using TeleForm Desktop version 9.1 (TeleForm, Verity Inc., Sunnyvale, CA). Mortality data were obtained from the National Death Register. This was possible only for patients with known identity (social security number) – 626/750 (83%) of the patients discharged alive from ambulances, 776/801 (97%) of the patients discharged from the outpatient clinic: in total, 1402/1551 (90%) of the patients discharged alive from pre-hospital treatment.

### Classification

The toxic agent was defined as the substance supposed to be most toxic in the amount taken. This classification was based on information from the patients or companions, clinical observations, and, if applicable, findings at the scene of the overdose. No toxicological testing was undertaken in the pre-hospital setting. In order to make the pre-hospital study form simple, only one main suspected agent was recorded (except in hospitals). An evaluation of the consistency of the substance determination between the treatment levels of transferred cases revealed that the receiving institutions (the outpatient clinic and the hospitals) and ambulance service considered the same agent as the most important in 78% of the cases. In 11%, the substance suspected in the ambulance was not considered as part of the poisoning at the other treatment levels, while in the rest (11%), another agent was considered as main agent in addition to the selected agent in the ambulance. The agents with the highest agreement between ambulance and the other levels were ethanol (92%), opioids (82%) and sedatives (81%). Various other kinds of medications had somewhat lower agreement.

Consciousness was classified according to the following scale: awake; somnolent (can be kept awake when stimulated); coma (responsive to painful stimuli); and deep coma (no response to painful stimuli). Coma and deep coma correspond to a Glasgow Coma Score < 8 [8]. Respiratory insufficiency was defined as a clinical need for respiratory support. Hypotension was defined as a systolic blood pressure below 85 mmHg in at least two subsequent measurements. Cardiac arrhythmias were registered from the cardiac monitoring screens or ECG. Conduction disturbances were classified as arrhythmias, but sinus tachycardia was not. Cardiac arrest was classified as such, not as arrhythmia. Because of the setting, the registration of complications in the ambulances and outpatient clinic was limited compared to the registration in hospital. Only respiratory depression and cardiac arrest were registered in the ambulances; in the outpatient clinic, hypotension and arrhythmia were also registered. Consciousness and clinical conditions were registered as seen in the outpatient clinic, independent of possible antecedent treatment in an ambulance.

### Statistics

Statistics were performed using SPSS software, version 15 (SPSS Inc., Chicago, Illinois). The Chi square test was used to compare frequencies, and comparisons of age were done using the Mann-Whitney U-test. A 5% significance level was used. As many p-values were calculated, significance was also corrected using the Holm-Bonferroni method, and the non-significance of apparently significant values was noted in the table legends. Logistic regression analyses were performed to analyse predictors of discharge versus transfer from pre-hospital levels. The variables entered into this analysis, selected primarily for their clinical importance, included gender, age, toxic agents, consciousness, respiratory depression and the use of antidote. Only variables with significant crude values were included in the multivariate analyses. Possible gender interactions with the other variables were searched for, but not found. Correlations between independent variables were calculated to ensure no correlation of 0.7 or more.

### Ethics

Treatment was given according to standard protocols, and the research was in accordance with the Helsinki Declaration. Permission for this study was obtained from The National Data Inspectorate and the Regional Ethics Committee. All data were stored anonymously, and Statistics Norway kept the link to social security numbers and names.

## Results

We recorded 2997 episodes of acute poisonings in 2298 individuals. The population in Oslo in 2004 was 521 886, of whom 428 198 were ≥ 16 years of age [9]. This gave an overall incidence of 5.4 per 1 000, but with no correction made for poisonings in persons living outside Oslo (the catchment area).

### Ambulances

In the ambulances, 1860 contacts were recorded (with nine double episodes on the same day), of which 1095/1860 (59%) were transferred to hospitals [525/1860 (28%)] or the outpatient clinic [570/1860 (31%)], 15/1860 (0.8%) died on scene, while 750/1860 (40%) were discharged and left at the scene without transfer (Additional file 1, Table 1).

Patients discharged alive from ambulances without transfer were more frequently male compared to those transferred [516/750 (69%)] versus [603/1095 (55%)] (Additional file 1, Table 1). The majority of the discharged patients had been poisoned with opiates [528/750 (70%)], while those transferred were frequently poisoned with pharmaceuticals [412/1095 (38%)] or ethanol [338/1095 (31%)]. Suspected opiate overdoses were discharged in 528/678 (78%) of cases (the 13 patients dead on scene excluded), as were ethanol poisonings in 171/509 (34%) of cases. Among all other suspected poisoning cases who survived, 51/658 (8%) were discharged. From all suspected opiate poisonings treated in ambulances, coma was present in 352/691 (51%). Coma was more frequent among those later discharged alive [265/750 (35%)] than those transferred [220/1095 (20%)]. Among those discharged after being comatose, 263/265 (99%) had opiate poisoning (reversed by naloxone in 255/263 (97%) of cases), while comas among the transferred were due to miscellaneous agents, with opiates suspected in only 76/220 (35%) of cases. From the patients with initial respiratory depression or respiratory arrest (lethal cases excluded), 275/380 (72%) were left at the scene, of whom 273/275 (99%) were assessed to have taken opioids and 259/275 (94%) received naloxone. In all, naloxone was given to 507/1860 (27%) patients, of whom 140/507 (28%) were transferred.

### Outpatient clinic

In the outpatient clinic, 956 contacts were registered (with two double episodes on the same day), of which 155/956 (16%) were transferred to hospital and 801/956 (84%) were discharged after treatment or observation in the clinic (Additional file 2, Table 2). There were no deaths. Patients were brought to the outpatient clinic by ambulance alone [453/956 (47%)]; by police alone or in cooperation with the ambulance [174/956 (18%)]; and by others or by themselves [329/956 (34%)]. Ethanol was the most common toxic agent [472/956 (49%)], followed by opiates [207/956 (22%)] and sedatives [91/956 (10%)]. 124/956 (13%) were comatose. Patients brought to the outpatient clinic by the police were more frequently comatose [42/174 (24%)] than patients with antecedent treatment in an ambulance [45/453 (10%)] or patients brought by other means [37/329 (11%)]; p < 0.001. Antidotes were given to 55/956 (6%), while 842/956 (88%) received no antidote or other specific treatment.

The majority of those discharged were poisoned with ethanol [439/801 (55%)] or opiates [188/801 (24%)], while those transferred were mainly poisoned with pharmaceuticals [64/155 (41%)] or ethanol [33/155 (21%)]. Cases of suspected opiate overdose were discharged in 188/207 (91%) of cases, while cases of ethanol poisoning were discharged in 439/472 (93%) of cases. In contrast to the ambulance patients, coma was less frequent in the discharged group [80/801 (10%)] than in the transferred group [44/155 (28%)], p < 0.001. Among those discharged after being comatose, 40/80 (50%) had ethanol poisoning and 27/80 (34%) opiate poisoning, while coma among the transferred cases was due to miscellaneous agents (ethanol in 13/44 (30%) and opiates in 10/44 (23%)). Among the ethanol poisoning cases discharged from the outpatient clinic, 424/439 (97%) received no specific treatment, only observation.

### Predictors for discharge versus transfer from pre-hospital settings

Logistic regression analyses were performed to study predictors of discharge versus transfer from the pre-hospital setting (Additional file 3, Table 3). Cases of poisoning with toxic agents other than opiates and ethanol were more commonly transferred than discharged, and toxic agents could therefore be merged into ethanol, opiates and other agents. For both pre-hospital service levels, adjusted odds ratios revealed no gender effect, and opiate and ethanol poisonings remained predictors with a high impact on discharge. Opiate poisonings were in particular a strong predictor for discharge from the ambulance (OR 38.8). Severe clinical conditions entailed transferral, but a significant high odds ratio remained for respiratory depression in the patients treated in ambulances. Age was not a significant predictor in the outpatient clinic patients, and was not included in the multivariate analysis of this group. Among the ambulance patients, those aged 50–69 years were less frequently discharged compared to patients younger than 30. When the multivariate analysis of the ambulance patients was done by age (for each age category separately), the predictive effect of opiates was greatest among the 30–49 year olds [OR = 68.9 (95% CI 35.8–132.9)] and in patients younger than 30 years [OR = 25.6 (95% CI 13.2–50.0)]. The predictive effect of ethanol was greatest among patients aged above 70 years [OR = 16.0 (95% CI 1.5–165.9)] and those aged 50–69 years [OR = 8.6 (95% CI 3.3–22.7)].

### Comparison of patients discharged from ambulances, the outpatient clinic and hospitals

**Figure 2:**
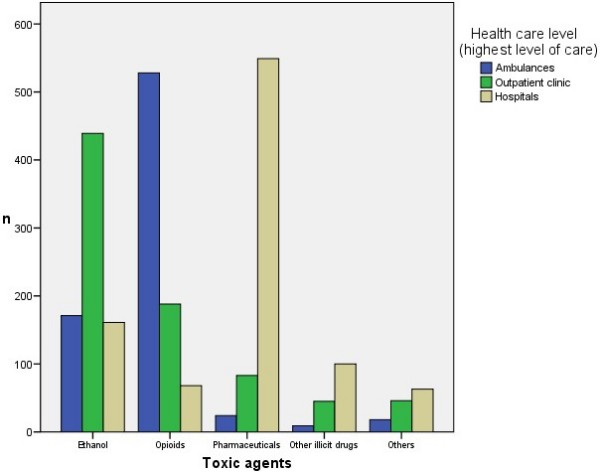
Suspected toxic agents in acute poisonings discharged from different levels of health care.

The patients who were discharged from pre-hospital health care settings (without further transfers) and patients treated in hospitals are compared in additional file 4, Table 4. In hospitals, 941 poisoning episodes were registered: the details of this group are published elsewhere [6,7]. The ambulance episodes consisted of slightly younger patients (median 34 years) than the outpatient clinic episodes (median 37 years, p = 0.002) and those in hospitals (median 36 years, p = 0.001). Gender differed between the pre-hospital and hospital settings: male patients dominated both ambulance and outpatient clinic episodes, with 69% in both, while only 46% of the hospitalized episodes were male patients (p < 0.001). The main toxic agents differed substantially (Figure 2 and Additional file 4, Table 4). In those treated in the ambulance only, 265/750 (35%) were comatose, which was significantly greater than the 80/801 (10%) comatose patients in those receiving their highest level of health care in the outpatient clinic (p < 0.001) and the 221/941 (23%) in hospitals (p < 0.001). Respiratory depression occurred more frequently in those treated in the ambulance only [275/750 (36%)] than those treated in the hospitals [67/941 (7%)], p < 0.001, which again was more frequent than in the outpatient clinic [35/801 (4%)], p < 0.02. Naloxone was given to 367/750 (49%) cases treated in ambulances (the only ambulance antidote), and naloxone or flumazenil were given to 34/801 (4%) patients in the outpatient clinic and 255/941 (27%) in the hospitals, p < 0.001 for all.

### Case characteristics within agent types

Of all opiate poisonings, 528/784 (67%) were treated in ambulances only, 188/784 (24%) in the outpatient clinic as the highest level of care, and 68/784 (9%) in the hospitals (Figure 2). 553/784 (71%) were male, and median age was 33 years. Of all ethanol poisonings, 439/771 (57%) were treated in the outpatient clinic as the highest level of care, 171/771 (22%) in the ambulances only, whereas 161/771 (21%) in the hospitals. 530/771 (69%) were males; median age 43 years. Pharmaceutical poisonings were mainly treated in hospitals [549/656 (84%)]; 227/656 (35%) were males; median age 37 years. In the paracetamol subgroup, 102/128 (80%) were females; median age 30 years. The youngest group was that with illicit drugs other than opiates, with a median age of 27 years; 117/154 (76%) males.

### One-week mortality after pre-hospital discharge

Fifteen patients died during ambulance treatment, while none died in the outpatient clinic. Social security numbers were known for 1402/1551 (90%) of the patients discharged from the pre-hospital levels [630/716 (88%) of the opiates], making a check against the National Death Register possible. One patient died of a new heroin overdose two days after the index episode when he was discharged from the ambulance; the antecedent episode entailed coma and respiratory depression reversed by naloxone. The forensic medicine record verified that this death was due to a new overdose, not complications after the index episode. There were no other deaths during the first week after the poisoning cases discharged from ambulances or the outpatient clinic.

## Discussion

The majority of poisoned patients treated by ambulance personnel and in the outpatient clinic were discharged without referral to higher treatment levels. These poisonings were mostly due to opiate overdoses and ethanol poisoning. Patients discharged from the ambulances were often severely poisoned regarding consciousness and respiratory depression or arrest, while those discharged from the outpatient clinic were less often comatose and less often had respiratory depression. Male patients dominated the pre-hospital discharged cases, while female patients were in the majority among the hospitalized, where pharmaceuticals were the most common toxic agents. One death due to a new overdose was recorded among the pre-hospital discharged in the first week after the poisoning.

Our findings of significant male dominance in the pre-hospital setting are supported by a previous study in Oslo, reporting 77% male patients [10], and by studies of pre-hospital opiate overdoses from Australia (67–77% males) [11,12] and Austria (66% males) [13]. In our study, female patients were in the slight majority among the hospitalized. Gender differences regarding toxic agents are often reported, with female patients poisoned with pharmaceuticals and male patients poisoned with non-pharmaceutical agents [14], which influences the gender distribution in the hospitals [6]. Studies on deliberate self-poisonings, which are more frequently admitted to hospital, show female majorities, with 66% in a Greek study [15] and 56%–66% in a Norwegian study [16]. Although discharging males more than females from the pre-hospital setting, the multivariate logistic regression analyses showed that gender was not an independent predictor for discharge versus transferral. It also revealed that the agent taken combined with the clinical severity were the key factors for patient flow through the system. This can probably be explained by opiate overdoses being potentially reversible on site, and ethanol poisoning as a transient, most often uncomplicated situation. Furthermore, many of the poisonings by medications need more thorough observation and treatment – which often implies hospitalization.

Although opiate overdoses often present with pronounced respiratory depression and altered consciousness, reversal by naloxone makes it possible for the patients to refuse further treatment, or further treatment is considered unnecessary. Norwegian law does not permit treatment against a person's will, except in suicidal or psychotic patients. The majority of such overdoses are not assessed as suicidal, but obviously, this evaluation is difficult on site. The situation in the outpatient clinic was quite different from that in the ambulances. Most patients were brought by ambulance or were able to be transported by other means. Ambulance personnel who decided to transport the patient to the outpatient clinic instead of the hospitals have made an evaluation based on the patient's clinical condition, resulting in the selection of probably less-affected patients compared to the remaining population. The outpatient clinic had the opportunity to observe patients for some hours, and fewer needed admission to hospital.

The present study reports a high proportion of opiate overdoses not transferred to a higher treatment level after ambulance treatment (78%) and outpatient clinic treatment (93%). Previously, 85% of opiate overdoses treated with naloxone were reported as not being transferred from ambulances in Oslo [10] (this subgroup comprised 72% in the present study). From most other countries, lower numbers were reported, but with large variations. In a Californian study, 166/609 (27%) were not transferred from the ambulances, and 12/442 (2.7%) were admitted from the Emergency Department (ED) [3]. Two studies from Austria reported 59/308 (19%) [13] and 295/1087 (27%) [17] not transferred, while an Italian study reported 52/124 (42%) not transferred to hospital [18]. However, an Australian study reported 324 (84%) not transferred of 388 naloxone-responders [19]. The higher proportion of non-transferred opiate overdoses in Oslo compared to most other studies is probably not due to differences in severity. It could reflect a more liberal tradition to either accept the wish not to be transported to hospital or to consider further referral as unnecessary. An Australian study across different jurisdictions showed that differences in overdose management, e.g. naloxone dosing, may affect the transportation and hospitalization rates [12].

Delayed complications, such as recurrence of opiate toxicity and occurrence of heroin-related non-cardiogenic pulmonary oedema (NCPE), are important in the assessment of whether patients were allowed to refuse transfer and observation. The half-life of naloxone is shorter than that of many opioids. Recurrence has been found in several studies, ranging from 31% to all admitted opiate overdoses [2,20]. The procedure in Oslo is to give one IM dose of naloxone before the IV doses, in order to prolong the naloxone effect. This study has no data on recurrence, as 78% of opiate overdoses treated in ambulances were not transferred. NCPE is found in about 2% of opioid overdoses treated in EDs [21,22], and is most frequently evident on arrival or within an hour. A study of pre-hospital heroin overdoses found pulmonary oedema in 0.8% [18]. In our study, 12% of the hospitalized opiate overdose cases (1% of all opiate overdose cases in the study) were mechanically ventilated [7], and presumably some of this was due to pulmonary oedema. As a result of delayed problems, recommendations for the observation time for an apparently uncomplicated opioid overdose reversed by naloxone have been made, but vary between one [23,24], two to three [22,25], and up to eight hours [26]. However, studies on opioid toxicity recurrence and NCPE are mainly performed in ED- or hospitalized patients, which are selected populations compared to patients not transferred from ambulance. Nevertheless, one has to assume that there may be a risk associated with the limited observation time offered by ambulance personnel when patients refuse further treatment. We have no data on the length of observation made by ambulance personnel, but a previous study in Oslo reported a mean observation time of 8 minutes (range 1–30 minutes) [10].

Drug abusers are difficult to follow up. Consequently, it is challenging to study complications other than death and readmissions among pre-hospital-treated overdoses. A study of early discharge of opiate overdose cases from an ED showed no readmissions or deaths [22], and studies on opiate overdose cases not transferred from ambulances found no subsequent life-threatening events [2] and no deaths due to the overdose [1]. This corresponds with our findings of only one death (which was due to a new overdose) in the first week after the poisoning among patients with known identity: 91% of all (88% of opiate) poisonings treated outside hospital.

Ethanol poisoning cases were the other large group of patients not transferred from the ambulances or outpatient clinic, most of which were observed in the outpatient clinic. In this study, 21% of ethanol poisoning cases were transferred to hospital from the outpatient clinic, which is more than the 12% admitted from the ED in an American register study of equivalent poisonings [27]. The Oslo Emergency Ward represents a lower treatment level than most hospital-based EDs, and hence transfers more patients to hospital. Ethanol poisoning is usually not life threatening. However, beside complications due to impaired consciousness, there is a risk of cardiac arrhythmias with heavy drinking [28]. A recent study of patients with high serum ethanol levels showed ECG changes associated with increased risk of arrhythmias [29]. Such risks should be taken into account when assessing ethanol-intoxicated patients. However, when in a stable clinical condition, early discharge of these patients is less controversial compared to in other poisonings.

In addition to the risk of complications, one can advocate for longer observation to channel patients into anti-addiction therapy. A French study showed that 80% of acute ethanol poisonings had elevated γ-glutamyltransferase and carbohydrate-deficient transferrin values, indicating harmful alcohol consumption over some time, suggesting that these patients should be offered treatment for alcoholism [30].

### Strengths and limitations

In spite of prospective inclusion and thorough follow-up of participating institutions in this study, patients suitable for inclusion might have been missed. Transferred cases not included in the study at the receiving institutions (lost to follow-up) may weaken the comprehensiveness of the study. On the other hand, the retrospective random check of a selection of this group revealed that failure to include at the receiving institution only account for about 20% of these patients. Most important is that the ambulance personnel have registered whether their patients were discharged after treatment or transported to a higher health care level, and this makes the basis for the results that are presented.

Not knowing the social security numbers of 124 of those discharged from ambulances and 25 from the outpatient clinic weakened the one-week mortality analysis. Nevertheless, although not covering the entire population, the mortality check of the 1404 poisonings treated out-of-hospital is valuable.

While additional agents were registered in hospitals, only one main toxic agent was registered for each patient in the pre-hospital setting. This was done for simplicity, to ensure the completeness of the registration in the pre-hospital setting, where time-pressure and the working conditions make the completing of a complex study form difficult. Poisoning with multiple toxic agents is common, and in the hospital population, multiple agents were suspected in 63% of patients [6]. A previous study of opiate overdoses in Oslo reported suspected additional agents in half of the cases [10], and a study of fatal poisonings among drug addicts in Scandinavia revealed a mean number of 2.4 toxic substances [31]. The clinical diagnosis of a particular toxic agent may be difficult and the precision of determining toxic agents may therefore be questioned, as confirmation by laboratory tests was not possible in the vast majority of the poisonings in this study. However, data was strengthened by the consistency of the evaluation of the main toxic agent in the ambulance service and the higher treatment levels of 78%, whereas only 11% of the cases were considered completely different.

The clinical condition of patients in the different treatment levels were not directly comparable as many of the patients in both the outpatient clinic and in the hospitals received antidote from the ambulance personnel before arrival, and hence presented less severe symptoms than in the ambulances. However, the registered status reflects the actual situation in the different institutions.

## Conclusion

Poisoned patients were often discharged from pre-hospital treatment levels. These poisonings were more often due to opiate and alcohol abuse than in the hospitalized patients, and more than two-thirds were male patients. Patients discharged from ambulances were often severely poisoned as regards consciousness and respiratory depression, whereas patients discharged from the outpatient clinic were less often comatose and less often had respiratory depression. Almost half of those discharged from an ambulance received an antidote. The pre-hospital treatment of these poisonings appears safe with regard to short-term mortality.

## Competing interests

The authors declare that they have no competing interests.

## Authors' contributions

FH structured the data, performed the statistical analyses and drafted the manuscript. KEH participated in the planning of the study and coordinated the collection of data. MAB participated in the collection of data and worked with the data files. AKN coordinated the outpatient clinic part of the study. JCPF coordinated the ambulance part of the study. OE participated in the design of the study and supervised the work. DJ conceived the study and supervised the work. All authors participated in revising the manuscript, and have read and approved the final version.

## Pre-publication history

The pre-publication history for this paper can be accessed here:



## Supplementary Material

Additional file 1 **Table 1, acute poisonings treated by the ambulance service.** Click here for file

Additional file 2 **Table 2, acute poisonings treated in outpatient clinic.** Click here for file

Additional file 3 **Table 3, predictors for discharging patients from pre-hospital levels.** Click here for file

Additional file 4 **Table 4, highest level of health care.** Click here for file
