# Supplementary material for: Pre-hospital treatment of acute poisonings in Oslo
Source: BMC Emerg Med. 2008 Nov 24;8:15. doi: 10.1186/1471-227X-8-15 (PMC2605443; doi:10.1186/1471-227X-8-15)
Supplement: Additional file 1 — Table 1, acute poisonings treated by the ambulance service. [file 1471-227X-8-15-S1.pdf]

Table 1. Acute poisonings treated by the ambulance service

		All ambulance patients		Transferred to hospital or outpatient clinic		Died on site		Discharged from ambulance without transfer		p-value (transferred vs. discharged)
		n	%	n	%	n	%	n	%	
Age (years)	Median	35		37		36		34		< 0.001[†]
	IQR	27–46		27–48		32–44		27–42		
Gender	Male	1130	(61)	603	(55)	11	(73)	516	(69)	< 0.001
	Female	730	(39)	492	(45)	4	(27)	234	(31)	
Main agent	Opiates	691	(37)	150	(14)	13	(87)	528	(70)	< 0.001
	Ethanol	509	(27)	338	(31)			171	(23)	< 0.001
	Sedatives	196	(11)	183	(17)			13	(2)	< 0.001
	Other illicit drugs	86	(5)	77	(7)			9	(1)	< 0.001
	Antidepressants	71	(4)	68	(6)			3	(0.4)	< 0.001
	Paracetamol	63	(3)	58	(5)			5	(1)	< 0.001
	Neuroleptics	4	(0.2)	4	(0.4)			0	(0)	0.1
	Other agents	240	(13)	217	(20)	2	(13)	21	(3)	< 0.001
Consciousness	Awake	934	(50)	602	(55)			332	(44)	< 0.001
	Somnolent	426	(23)	273	(25)			153	(20)	0.02[††]
	Comatose	181	(10)	103	(9)			78	(10)	0.48
	Deeply comatose	319	(17)	117	(11)	15	(100)	187	(25)	< 0.001
Complications	Respiratory depression	306	(17)	91	(8)			215	(29)	< 0.001
	Respiratory arrest*	74	(4)	14	(1)			60	(8)	< 0.001
	Cardiac arrest**	2	(0.1)	2	(0.2)			0	(0)	NA
	Dead***	15	(1)	–	(0)	15	(100)	–	(2)	NA
Antidotes	Naloxone	507	(27)	140	(13)	[†††]		367	(49)	< 0.001
Total		1860	(100)	1095	(100)	15	(100)	750	(100)	

IQR = Inter-quartile range
*Respiratory arrest without cardiac arrest
**With ROSC = Return of spontaneous circulation
***The two patients who survived cardiac arrest during ambulance treatment died later in hospital
[†]Mann–Whitney U-test
[††]Not significant when the whole table is corrected for multiple calculations using the Holm–Bonferroni method
[†††]All patients found in cardiopulmonary arrest, three received prolonged resuscitation, none received antidote
NA = not applicable
