# Supplementary material for: Pre-hospital treatment of acute poisonings in Oslo
Source: BMC Emerg Med. 2008 Nov 24;8:15. doi: 10.1186/1471-227X-8-15 (PMC2605443; doi:10.1186/1471-227X-8-15)
Supplement: Additional file 2 — Table 2, acute poisonings treated in outpatient clinic. [file 1471-227X-8-15-S2.pdf]

Table 2. Acute poisonings treated in outpatient clinic

		All outpatient clinic patients		Transferred to hospital		Discharged from outpatient clinic without transfer		p-value (transferred vs. discharged)
		n	%	n	%	n	%	
Age (years)	Median	37		36		37		0.10*
	IQR	26–46		26–44		26–47		
Gender	Males	647	(68)	92	(59)	555	(69)	0.02†
	Females	309	(32)	63	(41)	246	(31)	
Main agents	Ethanol	472	(49)	33	(21)	439	(55)	< 0.001
	Opiates	207	(22)	19	(12)	188	(24)	0.002
	Sedatives	91	(10)	34	(22)	57	(7)	< 0.001
	Other illicit drugs	59	(6)	14	(9)	45	(6)	0.11
	Paracetamol	22	(2)	15	(10)	7	(1)	< 0.001
	Neuroleptics	19	(2)	8	(5)	11	(1)	0.002
	Antidepressants	8	(1)	4	(3)	4	(1)	0.01†
	Other agents	78	(8)	28	(18)	50	(6)	< 0.001
Consciousness‡	Awake	322	(34)	48	(31)	274	(34)	0.44
	Somnolent	510	(53)	63	(41)	447	(56)	0.001
	Comatose	112	(12)	35	(23)	77	(10)	< 0.001
	Deeply comatose	12	(1)	9	(6)	3	(0.4)	< 0.001
Complications‡	Respiratory depression	53	(6)	18	(12)	35	(4)	< 0.001
	Arrhythmias	9	(1)	2	(1)	7	(1)	0.62
	Hypotension	22	(2)	10	(7)	12	(2)	< 0.001
	Cardiac arrest	0	(0)	–	–	–	–	NA
	Dead	0	(0)	–	–	–	–	NA
Activated charcoal		13	(1)	7	(5)	6	(1)	< 0.001
Antidotes	Naloxone or flumazenil	55	(6)	21	(14)	34	(4)	< 0.001
Total		956	(100)	155	(100)	801	(100)	

IQR = Inter-quartile range
*Mann–Whitney U-test
†Not significant when the whole table is corrected for multiple calculations by the Holm–Bonferroni method
‡As presented in the outpatient clinic, independent of possible antecedent treatment in the ambulance
