# Supplementary material for: Pre-hospital treatment of acute poisonings in Oslo
Source: BMC Emerg Med. 2008 Nov 24;8:15. doi: 10.1186/1471-227X-8-15 (PMC2605443; doi:10.1186/1471-227X-8-15)
Supplement: Additional file 3 — Table 3, predictors for discharging patients from pre-hospital levels. [file 1471-227X-8-15-S3.pdf]

Table 3. Predictors for discharging patients from pre-hospital levels without further transferral: logistic regression

From the ambulances		n	Crude effect OR	95% CI	p-value	Adjusted effect OR	95% CI	p-value
Gender	Male	1119	Reference category					
	Female	726	**0.6**	0.5–0.7	< 0.001	**0.9**	0.7–1.1	0.31
Toxic agents	Other agents	658	Reference category					
	Ethanol	509	**6.0**	4.3–8.5	< 0.001	**5.9**	4.1–8.3	< 0.001
	Opiates	678	**41.9**	29.9–58.8	< 0.001	**38.8**	25.4–59.1	< 0.001
Consciousness	Awake	934	Reference category					
	Somnolent	426	**1.0**	0.8–1.3	0.9	**0.4**	0.3–0.6	< 0.001
	Comatose	181	**1.4**	1.0–1.9	0.06	**0.2**	0.1–0.3	< 0.001
	Deeply comatose	304	**2.9**	2.2–3.8	< 0.001	**0.1**	0.1–0.3	< 0.001
Respiratory depression or arrest		380	**5.5**	4.3–7.0	< 0.001	**3.0**	1.8–4.7	< 0.001
Antidote	Naloxone	507	**6.5**	5.2–8.2	< 0.001	**2.0**	1.2–3.2	0.005
Age	<30	582	Reference category					
	30-49	875	**1.1**	0.9-1.4	0.4	**0.8**	0.6-1.1	0.15
	50-69	266	**0.4**	0.3-0.6	<0.001	**0.6**	0.4-0.9	0.009
	>70	55	**0.6**	0.3-1.1	0.07	**1.1**	0.6-2.3	0.75
	Age missing	67	**2.5**	1.5-4.3	0.001	**3.2**	1.6-6.2	0.001
	Total*	1845						

From the outpatient clinic		n	OR	95% CI	p-value	OR	95% CI	p-value
Gender	Male	647	Reference category					
	Female	309	**0.6**	0.5–0.9	0.02	**0.8**	0.5–1.3	0.3
Toxic agents	Other agents	277	Reference category					
	Ethanol	207	**7.9**	5.1–12.1	< 0.001	**8.1**	5.1–12.9	< 0.001
	Opiates	472	**5.9**	3.4–10.0	< 0.001	**12.6**	6.3–25.0	< 0.001
Consciousness	Awake	322	Reference category					
	Somnolent	510	**1.2**	0.8–1.9	0.3	**0.8**	0.5–1.3	0.3
	Comatose	112	**0.4**	0.2–0.6	< 0.001	**0.2**	0.1–0.4	< 0.001
	Deeply comatose	12	**0.1**	0.0–0.2	< 0.001	**0.04**	0.0–0.2	< 0.001
Respiratory depression		53	**0.3**	0.2–0.6	0.001	**0.3**	0.2–0.8	0.008
Antidote	Naloxone	55	**0.3**	0.2–0.5	< 0.001	**0.5**	0.3–1.2	0.1
	Total	956						

OR = Odds ratio for being discharged from pre-hospital levels
Adjusted effect: multivariate analysis including all variables shown in the tables, separately analysed for the ambulance and outpatient clinic patients
*Patients who died in the ambulances were excluded (n = 15)
