# Supplementary material for: Pre-hospital treatment of acute poisonings in Oslo
Source: BMC Emerg Med. 2008 Nov 24;8:15. doi: 10.1186/1471-227X-8-15 (PMC2605443; doi:10.1186/1471-227X-8-15)
Supplement: Additional file 4 — Table 4, highest level of health care. [file 1471-227X-8-15-S4.pdf]

**Table 4. Highest level of health care in 2492 episodes of acute poisoning**

| | Ambulance[†] | | Outpatient clinic | | Hospitals | | Odds ratio Ambulance vs hospital | | Odds ratio Outpatient clinic vs hospital | |
|---|---|---|---|---|---|---|---|---|---|---|
| | n | % | n | % | n | % | OR | 95% CI | OR | 95% CI |
| Age (median years) | 34 | | 37 | | 36 | | | | | |
| Males | 516 | (69) | 555 | (69) | 430 | (46) | 2.6 | 2.1–3.2 | 2.7 | 2.2–3.3 |
| Females | 234 | (31) | 246 | (31) | 511 | (54) | 0.4 | 0.3–0.5 | 0.4 | 0.3–0.5 |
| Opiates | 528 | (70) | 188 | (23) | 68 | (7) | 30.5 | 22.8–40.9 | 3.9 | 2.9–5.3 |
| Ethanol | 171 | (23) | 439 | (55) | 161 | (17) | 1.4 | 1.1–1.8 | 5.9 | 4.7–7.3 |
| Sedatives | 13 | (2) | 57 | (7) | 242 | (26) | 0.05 | 0.0–0.1 | 0.2 | 0.2–0.3 |
| Other agents | 21 | (3) | 50 | (6) | 125 | (13) | 0.2 | 0.1–0.3 | 0.4 | 0.3–0.6 |
| Other illicit drugs | 9 | (1) | 45 | (6) | 100 | (11) | 0.1 | 0.1–0.2 | 0.5 | 0.3–0.7 |
| Paracetamol | 5 | (1) | 7 | (1) | 116 | (12) | 0.05 | 0.0–0.1 | 0.06 | 0.0–0.1 |
| Antidepressants | 3 | (0.4) | 4 | (0) | 65 | (7) | 0.05 | 0.0–0.2 | 0.07 | 0.0–0.2 |
| Neuroleptics | 0 | (0) | 11 | (1) | 64 | (7) | | | 0.2 | 0.1–0.4 |
| Awake | 332 | (44) | 274 | (34) | 436 | (46) | 0.9 | 0.8–1.1 | 0.6 | 0.5–0.7 |
| Somnolent | 153 | (20) | 447 | (56) | 284 | (30) | 0.6 | 0.5–0.7 | 2.9 | 2.4–3.6 |
| Comatose | 78 | (10) | 77 | (10) | 140 | (15) | 0.7 | 0.5–0.9 | 0.6 | 0.5–0.8 |
| Deeply comatose | 187 | (25) | 3 | (0) | 81 | (9) | 3.5 | 2.7–4.7 | 0.04 | 0.0–0.1 |
| Antidote* | 367 | (49) | 34 | (4) | 255 | (27) | 2.6 | 2.1–3.2 | 0.1 | 0.1–0.2 |
| Total | 750[†] | (100) | 801 | (100) | 941 | (100) | | | | |

[†]15 patients dead on site not included

*The antidote was solely naloxone in ambulances, while only naloxone and flumazenil were included from outpatient clinic and hospitals
